# Terahertz graphene-based tunable capacitance metamaterials with ultra-high amplitude modulation depth

**DOI:** 10.1038/s41377-025-02037-z

**Published:** 2025-10-02

**Authors:** Zi-Jun Guo, Geng-Bo Wu

**Affiliations:** State Key Laboratory of Terahertz and Millimeter Waves, Department of Electrical Engineering, City University of Hong Kong, Kowloon Tong, Hong Kong SAR, China

**Keywords:** Terahertz optics, Metamaterials

## Abstract

By combining substrate-side, phase-cancelling reflection with monolayer graphene reconfigured as nanoscale tunable lateral capacitors within metasurface unit cells, terahertz amplitude modulation exceeding 40 dB at around 2 THz with 30 MHz reconfiguration speed is demonstrated under solid-state, room-temperature conditions. The design provides a scalable and practical platform for high-speed, large-dynamic-range terahertz communications, real-time imaging, and programmable photonic circuits.

Terahertz modulators serve as essential tools for probing material structure and composition, and are also pivotal in applications such as high-speed wireless communication, real-time imaging, and on-chip optical computing^1^. Micro-electromechanical systems (MEMS)^2^, vanadium oxide (VO_2_)-based phase-change devices^3^, liquid crystal^4^ and liquid ion-gating of graphene^5^ have offered high modulation depths, but only at kilohertz speeds. Semiconductor high-electron-mobility transistors (HEMTs) are capable of gigahertz-frequency operation, but their utility in the terahertz regime is severely constrained by ohmic contact limitations and parasitic effects^6^. In a recent issue of Light: Science & Applications, Xia et al. report a graphene-based tunable capacitance metamaterial that achieves 100% amplitude modulation in the terahertz range with high reconfiguration speed^7^.

Conventional graphene-enabled metamaterial-based active modulators typically integrate a continuous graphene layer directly beneath capacitive unit cells, functioning as a variable resistor that actively dissipates energy from LC resonances, thereby constraining modulation efficiency^8^. Furthermore, the finite conductivity of graphene at the Dirac point fundamentally caps attainable modulation depth^9^. Xia et al. proposed an electrically reconfigurable capacitive metamaterial leveraging graphene, synergized with substrate-side (back) illumination to substantially enhance modulation depth and efficiency across the terahertz spectrum.

The first key innovation involves inserting an air gap into the graphene patch beneath the metallic antenna gap; this reconfigures monolayer graphene from its conventional “variable resistor” role into a nanoscale, voltage-tunable parallel-plate capacitor embedded within a brickwork metamaterial (Fig. 1). As the back-gate voltage is swept from 0 V to +50 V, the Fermi level in graphene shifts rapidly, inducing a pronounced variation in the capacitance *C*_gr_ within the equivalent circuit model. This effectively adds a voltage-tunable capacitor in parallel with the LC resonator instead of a lossy resistor. The modification nearly doubles the modulation efficiency: at 2.15 THz the reflectance can be continuously tuned from 100% to below 0.01%, corresponding to an amplitude extinction of 40.1 dB, while at 1.68 THz the extinction reaches 45.7 dB—far exceeding the <10 dB previously reported for graphene metasurfaces—all achieved using gate voltages between 0 and 50 V.

**Fig. 1:**
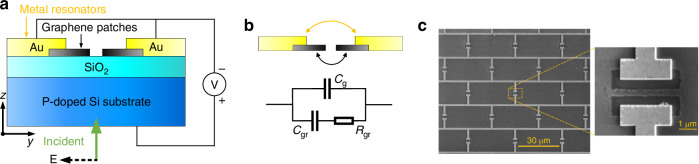
Schematics of the brickwork tunable capacitance modulator. **a** Cross-section view of the unit cell. **b** Equivalent circuit model of the capacitance metamaterial unit cell. **c** Fabrication prototype of the brickwork modulator. Figure was reproduced from ref. ^7^

The second key innovation involves modulating light incident from the back (substrate) side and reflected at the metasurface-air interface. Instead of illuminating the device from the air side, terahertz waves are emitted from the substrate side. This approach exploits the inherent 180° phase difference between the Fresnel reflection at the substrate-air interface and the reflection phase of the metasurface. By electrically tuning the graphene sheet conductance between 0.18 and 0.9 mS, destructive interference between these two reflected components drives the reflectance to near-zero. This mechanism delivers 100% amplitude modulation depth while circumventing the speed limitations inherent in quarter-wave resonance cavities^10^, Brewster-angle architectures^11^ or ion-gel-gated configurations^12^.

The fabrication sequence begins with a 4-inch, 525 ± 25 µm boron p-doped high-resistivity silicon substrate ( ≥ 100 Ω·cm) that is double-side coated with 300 nm silicon dioxide. The backside oxide is removed by reactive-ion etching, leaving the 300 nm SiO₂ gate dielectric only on the top surface. Monolayer graphene grown on Cu foil by chemical vapour deposition—commercially sourced for 2.15 THz devices and home-grown for 1.68 THz devices—is then wet-transferred onto the wafer. Electron-beam lithography followed by oxygen-plasma etching defines the graphene patches and the air gap inside each capacitive gap. A second electron-beam lithography step and thermal evaporation of 14 nm Ti / 156 nm Au complete the brickwork antenna arrays. This sequence avoids ionic liquids, Brewster-angle devices, phase-change films or complex back cavities, thereby eliminating moving parts, thermal hysteresis and high gate-voltage requirements—establishing a manufacturable route to large-area, room-temperature, high-speed terahertz devices.

Although record-breaking 40 dB amplitude extinction and 30 MHz reconfiguration speed have been achieved, challenges persist in the form of appreciable insertion loss and a narrow operational bandwidth. Suppressing radiative leakage, lowering graphene–metal contact resistance, and extending the modulation depth over a broad spectral window remain ongoing hurdles. As these technologies mature—from nanoscale tunable capacitors to substrate-side destructive-interference architectures—they are expected to rapidly evolve from laboratory prototypes into backbone infrastructure, ensuring terahertz links are not only high-speed and secure but also seamlessly woven into the fabric of the digital society.
